# Interfacial Contact Behavior between CNTs and AgNW with Molecular Dynamics Simulation

**DOI:** 10.3390/ma13061290

**Published:** 2020-03-12

**Authors:** Jianlei Cui, Huanhuan Mei, Jianwei Zhang, Zhengjie Fan, Jun Yang, Wenjun Wang, Hironori Tohmyoh, Xuesong Mei

**Affiliations:** 1 State Key Laboratory for Manufacturing Systems Engineering, Xi’an Jiaotong University, Xi’an 710049, China; mhhxjtu@stu.xjtu.edu.cn (H.M.); guangwei20120228@stu.xjtu.edu.cn (J.Z.); fanzhengjie@xjtu.edu.cn (Z.F.); softyj@mail.xjtu.edu.cn (J.Y.); wenjunwang@mail.xjtu.edu.cn (W.W.); 2 Department of Finemechanics, Tohoku University, Aoba 6-6-01, Aramaki, Aoba-ku, Sendai 980-8579, Japan

**Keywords:** contact behavior, carbon nanotubes, Ag nanowire, interfaces, MD simulation

## Abstract

The behavior at an interface between carbon nanotubes (CNTs) and silver nanowire (AgNW) could hardly be observed experimentally on an atomic scale, and the interaction is difficult to accurately calculate due to nanometer size effects. In this work, the contact behavior is studied with the molecular dynamics (MD) simulation, which indicates that the CNTs and AgNW can move towards each other to form aligned structures with their interfaces in full contact. In these different composite systems, nanotubes may either keep their form of an inherent cylindrical structure or completely collapse into the nanoribbons that can tightly scroll on the AgNW periphery while wrapping it in a core-shell structure. Thus, the atomic configuration evolution that is affected by the van der Waals (vdW) interaction is closely analyzed to assist the understanding of interfacial contact behavior.

## 1. Introduction

Carbon nanotubes (CNTs), including single-walled carbon nanotubes (SWNTs), double-walled carbon nanotubes (DWNTs), and multi-walled carbon nanotubes (MWNTs), have extraordinary properties due to their unique hollow tubular structures. They have unique hollow structures and exceptional electrical, thermal, mechanical and optical properties, showing limitless applications in the integrated circuit, flexible electronics, heterogeneous catalysis, and composite fields, among others. [1,2,3,4,5,6,7,8,9,10,11,12]. In the innovative exploration of the behaviors of CNTs, the pristine tubular structure is easily changeable under the influence of external factors. On the basis of the radial deformation of CNTs reported by Ruoff et al. [13], Chopra et al. [14] revealed, for the first time, the complete collapse behavior of SWNTs along tube length. They also found that some CNTs have localized minor deformation, such as kinks and bends in the radial direction. Additionally, studies of the radial collapse of SWNTs on macroscopic Cu and Al surfaces, both with molecular dynamics (MD) simulations, were conducted, respectively, by Yan et al. [15] and Xie et al. [16], which demonstrated that the potential application in the corrosion protection and scale inhibition fields. However, compared to macroscopic materials, nanomaterials have been known to possess a variety of unique physical and chemical properties based on their high surface-to-volume ratio and nanometer dimension, which is different from macroscopic materials. Thus, if the macroscopic material surfaces in contact with CNTs are replaced by nanowire (NW) surfaces, will similar behavior or other results occur? Thus, copper nanowire, the substitute for previous microscopic material, was adopted in the study of SWNT collapse behavior by Yan et al. [17] with the MD method. As the study showed, copper nanowire can induce nanotube collapse to produce core/shell composite nanowires through the self-scrolling behavior of collapsed SWNTs. Similarly, Chen et al. [18] applied the MD method to simulate the interaction between SWNTs and aluminum nanowires, and they revealed that nanowire can activate, guide, and stabilize the self-assembly of SWNTs to form a double-deck helix. Furthermore, Zhang et al. [19,20] studied the interaction between SWNTs and platinum, as well as the interaction between SWNTs and silver nanowire. Their finding suggested that nanowires can induce the self-assembly of SWNTs to form a shell-core structure. Likewise, we investigated the interfacial collapse behavior and atomic configuration evolution of SWNTs on metal nanowires with MD simulations [21,22]. Simultaneously, we were inspired to think about the questions like what would happen between MWNTs and nanowires and what can account for the differences in their behaviors in MWNT–NW and SWNT–NW systems. Additionally, considering the difficulty in experimentally observing interfacial behavior and accurately calculating the interaction on the nanometer scale, the contact behavior at the interface between CNTs and Ag nanowire (AgNW) is revealed with the molecular dynamics method.

## 2. Simulation Methods

This work was simulated under the force field of Condensed-phase Optimized Molecular Potentials for Atomistic Simulation Studies (COMPASS) in the Discover module based on the commercial software platform of Materials Studio (v7.0, Accelrys Software Inc., San Diego, CA, USA) [23,24,25], which can enable the accurate prediction for the behavior of the metal and nonmetal materials [26,27,28,29]. The system energy is the sum of the valence interaction, the cross-term interaction, and the nonbond interaction. The nonbond energy includes the vdW interaction, the Coulombic term, and hydrogen bond energy. In this simulation, because the values of Coulomb’s interaction and hydrogen bond interaction are both zero, the nonbond energy is equal to the vdW interaction. Thus, according to our previous MD studies on the interactions between CNTs and metal nanowires, the vdW interaction plays an essential role in their condensed-phase properties and behaviors [21,22]. Due to the size effect of nanomaterials, the non-periodic boundary was set on x, y and z directions of the CNT-Ag nanowire (CNT–AgNW) model. Before the simulations of interfacial contact behavior, the ‘Smart Minimizer’ function was first carried out to optimize atomic configurations, and the convergence criteria of 1000 kcal mol^−1^ Å^−1^ and maximum interactions of 5000 were set. Then, the optimized CNT–AgNW model was run under an NVT (N gives the number of atoms, V represents the volume, and T is the temperature) ensemble with the thermostat of Andersen. The atom-based summation method was used for vdW interactions, and the parameters of cutoff, spline width, and buffer width were, respectively, set to 9.50, 1.00, and 0.50 Å. In addition, the velocities of the atoms were set according to the Maxwell–Boltzmann distribution. In order to balance the fluctuation amplitude and stabilization time of system energy, according to our previous MD work [30,31,32], a simulation time of 1 ns with a 2 fs time step was set, and the integration method of velocity Verlet was chosen in the simulations. Finally, by storing the trajectories of all atoms, corresponding atomic configurations were output as needed.

## 3. Results and Discussion

**Figure 1:**
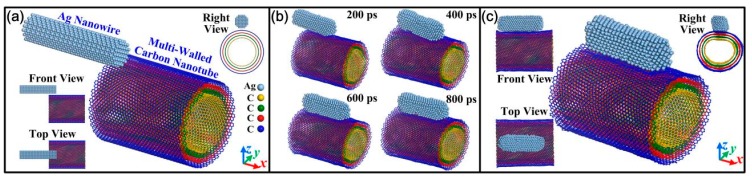
(**a**) Initial atomic configuration of the misaligned structure, (**b**) evolution of configuration, and (**c**) final configuration between the silver nanowire (AgNW) and multi-walled nanotube (MWNT) at 500 K.

Figure 1a gives a typical atomic model of a misaligned structure between AgNW and MWNT. Seen from its cross section, the MWNT was composed of four separated SWNTs, namely the metallic armchair (30, 30) nanotubes, the semiconducting chiral (40, 30) and (50, 30) nanotubes, and the metallic chiral (60, 30) nanotubes, respectively, from inside to outside. Since only interfacial contact behavior was revealed in this simulation, the AgNW of 8.0 nm in length and 2.0 nm in diameter was chosen based on the work load and operation time of the simulations. Then, the MWNT with a similar length of 8.116 nm, consisting of four separate walls with a wall separation of 3.347 Å, was also selected in order to avoid the influence of other factors. At the two ends of MWNTs, the dangling C atoms were unsaturated. Then, in the contact overlap area of MWNTs and AgNW, the dangling C atoms on the left end of the MWNT could attract the Ag atoms near them on the left with vdW interaction. Thus, at the interface between the AgNW and MWNTs in Figure 1b, they moved towards each other. When the time was 400 ps, they formed the aligned structure with their interfaces in full contact. In this state, the vdW force between the interfaces was at its maximum to induce structure change. However, in terms of the atomic configuration of the MWNT, they maintained the inherent concentric cylindrical structure (Video S1), which was obviously different from the reported deformation behavior of the SWNT on the surface of metal nanowires or metal substrates. In addition, though the surface morphology of the AgNW changed slightly, it was still in a solid state from its compact and regular atomic configurations at a relatively high temperature.

**Figure 2:**
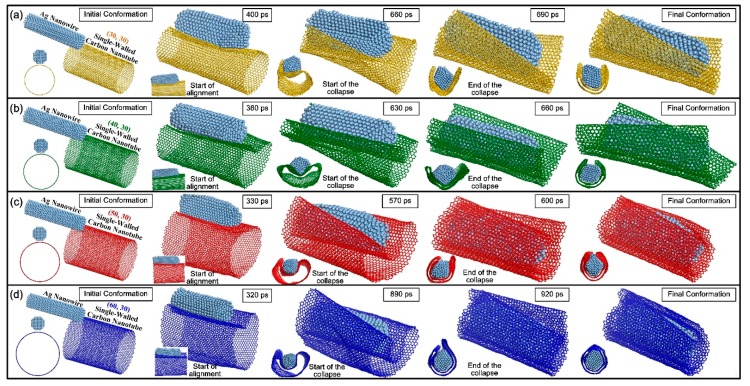
The evolution of atomic configurations between AgNW and single-walled nanotubes (SWNTs) with different chiralities of (**a**) (30, 30), (**b**) (40, 30), (**c**) (50, 30) and (**d**) (60, 30) at 500 K, respectively.

Simultaneously, in order to investigate the different contact behavior between the AgNW–MWNT system and the AgNW–SWNT system, the MWNT was decomposed into four separated SWNTs. The interfacial contact behavior between the AgNW and SWNTs was simulated accordingly, and Figure 2 demonstrates the evolution of atomic configurations. Apparently, although the four SWNTs differentiated in chiralities of (30, 30), (40, 30), (50, 30), and (60, 30), respectively, their diameters gradually increased with large differences. The increase in diameter could have affected the reduction in the number of unsaturated dangling C atoms on the left part of the SWNT close to the Ag atoms at their interface. The driving force to induce the movement of the SWNT also weakened accordingly. Thus, in terms of their movement, the aligned structures were eventually formed with the decreasing start time points of 400, 380, 330, and 320 ps in different AgNW–SWNT systems. When the AgNW was in full contact with SWNT (Videos S2–S5), the vdW force started to get stronger. The SWNT also began to suffer from the stronger attraction from AgNW. Furthermore, the collapse deformation also depended on the radial rigidity of the hollow nanotube. The SWNT with a larger diameter was weaker in its rigidity. Thus, as the SWNTs with chiralities of (30, 30), (40, 30) and (50, 30) were selected, the nanotube, started to collapse at the decreasing time points of 660, 630, and 570 ps. However, owing to the reduction in the number of interfacial C atoms, the larger the diameter was, the weaker attractive force from AgNW at the interface became. For this reason, although the (60, 30) SWNT had a weak radial rigidity due to its large diameter, the relatively weak vdW force could have hardly induced a quick nanotube collapse. Then, after a long relaxation, the (60, 30) nanotube started to collapse from 890 ps. Additionally, although the collapse starts and ends at different time points in different AgNW–SWNT systems, collapse duration is basically maintained in a very short range of 30 ps based on the π–π stacking effect. However, as their appearance changed, the SWNTs completely collapsed into nanoribbons in the shape of bilayer graphene-like structures. After that, they tightly scrolled on the AgNW periphery, wrapping it in a core-shell structures. Furthermore, if the length of the formed nanoribbons was close to the circumference of AgNW, and the curling and wrapping behavior could be seen as the process of converting the bilayer-graphene-like structure into a DWNT-like structure based on the final atomic configurations in the cross section.

**Figure 3:**
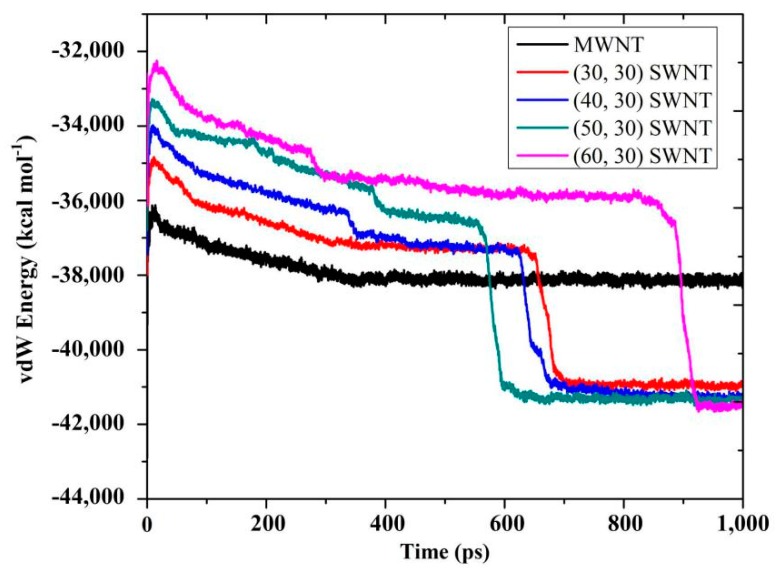
The relationship between van der Waals (vdW) energy and simulation time in an AgNW–MWNT system and different AgNW–SWNT systems at 500 K.

In order to study the mechanism of the aforementioned contact behavior differences in the AgNW–MWNT and AgNW–SWNT systems, Figure 3 shows the vdW energy curve with simulation time. Evidently, in all AgNW–SWNT systems, there was a sharp decrease in the energy evolution within a very short time. By combining with the evolution of atomic configurations in Figure 2, vdW force caused the collapse behavior of SWNTs on the AgNW surface on the premise that vdW energy could exceed the energy threshold of structural collapse of different SWNTs. Hence, before the energy rapidly decayed, the slow decreasing energy over time was related to the movement between the AgNW and the SWNTs at their interfaces. After the complete collapse of the SWNT, the slight adjustment of energy corresponded to the relaxation of atomic configurations for the energy minimization of the system. By contrast, in the AgNW–MWNT system, there was only a slow decrease in energy corresponding to their interfacial movement. This also shows that the vdW force at the interface was well below the collapse threshold based on the stable multi-layer structure of the MWNT.

**Figure 4:**
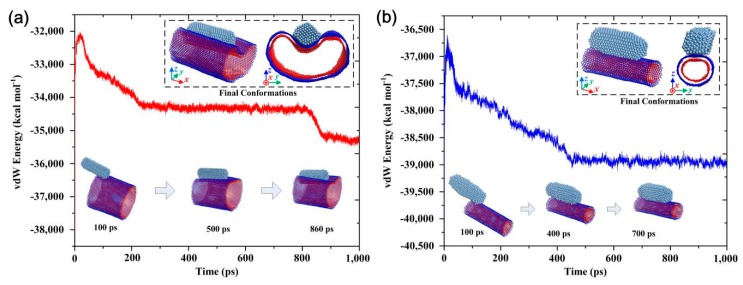
The relationship between vdW energy and simulation in (**a**) a large-diameter AgNW–DWNT (double-walled carbon nanotube) and (**b**) small-diameter AgNW–DWNT systems with illustrated atomic configurations in different times at 500 K.

The contact behavior and vdW effect in the AgNW–MWNT system also inspired us to investigate whether the collapse behavior could occur when the number of layers when down. Figure 4 shows the vdW energy evolution and atomic configurations between AgNW and a large-diameter DWNT with (50, 50) inner and (60, 50) outer nanotubes, as well as between AgNW and a small-diameter DWNT with (15, 15) inner and (25, 15) outer nanotubes. The interfacial motion behavior formed aligned structures based on the evolution of atomic configurations (Video S6). Unfortunately, collapse behavior did not happen. In the system of the AgNW and the large-diameter DWNT, the diameter of the outer (60, 50) nanotube was larger than that of the (60, 30) SWNT in Figure 2. Even if the AgNW was in full contact with the DWNT, fewer interfacial C atoms of the outer nanotube interacted with the AgNW. Then, the vdW force at the interface was smaller than that in the system of Figure 2d. Moreover, although the rigidity of the separated nanotubes of the DWNT was not as high as that of the (60, 30) nanotube, the rigidity of the whole DWNT was much greater. Thus, the vdW force made it hard to induce collapse. Only the large-diameter DWNT showed significant radial deformation. In Figure 4, it can be seen that, although there was a distinct step change in the vdW energy evolution curve, the energy difference did not reach its complete collapse threshold. Additionally, compared with the large-diameter DWNT, the small-diameter DWNT had better rigidity, which also made it difficult for the vdW force to induce collapse. It still retained the inherent cylindrical structure, and the contact behavior and vdW energy evolution were similar to the situation in Figure 1. Therefore, it can be concluded that without external force applied, MWNTs can maintain their structure well, while SWNTs are prone to collapse under the vdW energy at their interfaces.

## 4. Conclusions

In this study, the interfacial contact behavior at an CNT–AgNW interface was investigated with MD simulations. The AgNW and CNTs were able to move towards each other to form an aligned hybrid structure. In the AgNW–MWNT systems, nanotubes maintained their inherent concentric cylindrical structure, while in the AgNW–SWNT systems, SWNTs were prone to completely collapse into nanoribbons that could tightly scroll on the AgNW periphery, wrapping it to form core-shell structures. As analyzed, the interfacial contact behavior was seriously affected by vdW energy, as well as the collapse threshold of the CNTs. Hopefully, this study can provide an understanding of AgNW–CNT interfacial behavior and guidance for future applications, such as nanomotors, heterogeneous catalysis, and composites.

## Supplementary Materials

The following are available online at https://www.mdpi.com/1996-1944/13/6/1290/s1, Video S1: Interaction between MWNT and AgNW, Video S2: Interaction between (30-30) SWNT and AgNW, Video S3: Interaction between (40-30) SWNT and AgNW, Video S4: Interaction between (50-30) SWNT and AgNW, Video S5: Interaction between (60-30) SWNT and AgNW, Video S6: Interaction between DWNT and AgNW.
